# Supplementary material for: Active site specificity profiling datasets of matrix metalloproteinases (MMPs) 1, 2, 3, 7, 8, 9, 12, 13 and 14
Source: Data Brief. 2016 Feb 22;7:299–310. doi: 10.1016/j.dib.2016.02.036 (PMC4777984; doi:10.1016/j.dib.2016.02.036)
Supplement: Supplementary file 10 — Supplementary material [file mmc10.zip › WebPICS_hMMP1_T_1%/P1prime.html]

 

PICS results


|  |  |
| --- | --- |
| **P1prime\_C**  6 in 277 sites   2.2 %    effects > 10 perc. pnts.  (vice-versa in brackets)  P3prime\_T: 43.9 (15.4) |  |
| **P1prime\_M**  23 in 277 sites   8.3 %    effects > 10 perc. pnts.  (vice-versa in brackets)  P2\_S: 12.7 (11.7) |  |
| **P1prime\_V**  22 in 277 sites   7.9 %    effects > 10 perc. pnts.  (vice-versa in brackets)  P2\_M: 14.6 (32.1)   P1\_H: 10.7 (29.6) |  |
